# Ultrasound-Assisted Extraction Optimization of Proanthocyanidins from Kiwi (*Actinidia chinensis*) Leaves and Evaluation of Its Antioxidant Activity

**DOI:** 10.3390/antiox10081317

**Published:** 2021-08-21

**Authors:** Ji-Min Lv, Mostafa Gouda, Yan-Yun Zhu, Xing-Qian Ye, Jian-Chu Chen

**Affiliations:** 1 National-Local Joint Engineering Laboratory of Intelligent Food Technology and Equipment, Zhejiang Key Laboratory for Agro-Food Processing, Zhejiang Engineering Laboratory of Food Technology and Equipment, College of Biosystems Engineering and Food Science, Zhejiang University, Hangzhou 310058, China; lvjimin@zju.edu.cn (J.-M.L.); 11813043@zju.edu.cn (Y.-Y.Z.); psu@zju.edu.cn (X.-Q.Y.); 2 Department of Nutrition & Food Science, National Research Centre, Dokki, Giza 12422, Egypt

**Keywords:** proanthocyanidins, ultrasound-assisted extraction, kiwifruit leaves, extraction optimization, HPLC-QTOF-MS/MS, antioxidant potential

## Abstract

Using ultrasound (US) in proanthocyanidin (PA) extraction has become one of the important emerging technologies. It could be the next generation for studying the US mechnophore impact on the bioactive compound’s functionality. The objective of this study was to demonstrate the potential of US treatment on PAs extracted from kiwifruit (*Actinidia chinensis*) leaves, and to provide a comprehensive chemical composition and bioactivity relationship of the purified kiwifruit leaves PAs (PKLPs). Several methods like single-factor experiments and response surface methodology (RSM) for the four affected factors on US extraction efficiency were constructed. HPLC-QTOF-MS/MS, cytotoxicity analysis, and antioxidant activity were also demonstrated. In the results, the modeling of PA affected factors showed that 40% US-amplitude, 30 mL/g dry weight (DW) solvent to solid ration (S/S), and 70 °C for 15 min were the optimum conditions for the extraction of PAs. Furthermore, PKLPs exhibited significant radical scavenging and cellular antioxidant activity (*p* < 0.05). In conclusion, this study’s novelty comes from the broad prospects of using US in PKLP green extraction that could play an important role in maximizing this phytochemical functionality in drug discovery and food science fields.

## 1. Introduction

Kiwi (*Actinidia chinensis*) leaves are natural sources that are considered as a byproduct of kiwi fruit production and that contain a very high amount of natural bioactive phytochemicals that are generally considered as natural, safe extracts [1,2]. For instance, it has a high amount of polyphenols 189.39–440.71 mg GAE/g DW [3] that have a high bioactivity for human health (e.g., antioxidant and anti-tumor), due to their functional groups like hydroxyl groups on its phenolic rings [4]. In particular, proanthocyanidins (PAs) are a class of polyphenols that are divided into different types based on the hydroxylation patterns of their monomeric flavan-3-ols units [5], in which PAs oligo- or polymers are produced as an end product of the flavonoid biosynthetic pathway [6]. These phytochemicals are considered as offense and defense molecules because of their antioxidant [7], anticancer [8], antidiabetic [9], and antimicrobial activities [8]. Zheng et al. [10] reported that PAs are considered as a key ingredient that helps in the novel functional foods industry. For instance, they can inhibit the α-amylase enzyme activity in the phenolic-starch complexes. As a result, this reduces the starch digestibility, which has significant benefits for the delivery of functional molecules in humans [11].

Newly developed technologies are currently used for the extraction and structural analysis of PAs [12]. Regarding the emerging technologies for PA extraction, US has a significant impact on increasing the efficiency of plant phytochemicals, especially polyphenolic PAs [13]. For example, using US for extraction of PAs from different plant origins like grape seeds and knotweed rhizome bark at 40 °C for 15–20 min enhanced their extracted yields [14,15]. Boulatov [16] reported that the mechanochemical effects of the US results in overstretching of macromolecule polymers (like carbohydrate and protein chains) that lead to their fragmentation and release of phytochemicals. A comparison study of US-assisted and conventional extraction methods on tea leaves’ bioactive compounds showed that the breakdown of cells cytoarchitecture by US increased 20% of extracted bioactive phenolics [17]. In addition, Rashed et al. [18] reported that higher total phenolic content was extracted from *Lavandula pubescent* leaves using US compared to maceration extraction (ME).

For most plant leaves, the US acoustic cavitation can facilitate the flow of solvents into the plant cells and enhance the desorption of the bioactives from the matrix of solid samples. In addition, the formation of radicals during the optimum conditions of US process can increase the antioxidant activity of the extracted PAs through extension in the hydroxylation process [19]. This led to the fact that the optimum US conditions for various plant materials are maximizing the extracted yields and their bioactivities. In addition, US inhibits the hydrolyzing enzymes (e.g., α-glucosidase) that have a negative effect on flavonoids like PAs and their antioxidant functionality [20]. Collectively, the above information suggests a scope for optimization where maximum bioactivities could be obtained before the negative effects by heating or free radicals from the high US-amplitude. For instance, as a statistical technique, response surface methodology (RSM) has been proved as an effective tool for optimizing the process parameters that enable the reduction of the number of experimental trials and quantify the interactions between the multiple parameters [21]. Furthermore, several technologies, like high-performance liquid chromatography-Quadrupole Time of Flight Mass Spectrometry (HPLC-QTOF-MS/MS), and matrix-assisted laser desorption/ionization (MALDI), are recently validated as novel applicable methods for qualitative and quantitative analysis of PA structure conformation [12].

The use of bioactive PAs, as natural safe phytochemicals, in the prevention and treatment of different kinds of cancers has become one of the world’s hottest scientific areas [22,23]. Nutraceuticals derived from fruits, vegetables, and herbal leaves (kiwi, pomegranate, orange) generally have multi-targeted anticancer potential with negligible side-effects, thus making them ideal candidates for non-pharmacologic anticancer therapies, especially for colorectal cancer (CRC), which remains the fourth most common cause of cancer-related deaths worldwide [24,25]. HepG-2 and Caco-2 cell lines that related to CRC are wildly used to investigate the anticancer efficiency of the bioactive phytochemicals, especially PAs [26].

Therefore, this study aimed to evaluate the US impact on the extraction yield and chemical compositions of PAs extracted from kiwi leaves. Different US extraction conditions were evaluated to optimize the US conditions for PA extraction. Then, the optimum PA extract by using US was compared with its extract by ME for its anticancer and antioxidant functionality, thus providing evidence of the mechanochemical applicability of US as a green technology in the design of PA extraction while caring for their bioactive functionality. In addition, to evaluate the potential of using PAs as antioxidant and anticancer agents based on their unique chemical structures.

## 2. Materials and Methods

### 2.1. Chemicals and Materials

Acetone and ethanol that used in the part of extraction were purchased from Aladdin Reagent Co. Ltd. (Shanghai, China). AB-8 Macroporous resin was obtained from Solarbio Science and Technology Company (Beijing, China). Folin–Ciocalteu reagent was purchased from Aladdin Reagent Co., Ltd., Shanghai, China. Acetonitrile and formic acid of HPLC grade were purchased from Merck Chemicals (St. Louis, MO, USA). Deionized water was used. Foetal bovine serum (FBS), Dulbecc’s modified Eagle medium (DMEM), phosphate-buffered solution (PBS), penicillin/streptomycin (P/S), and methyl thiazolyl tetrazolium (MTT) were purchased from Life Technologies (Carlsbad, CA, USA).

**Figure 1:**
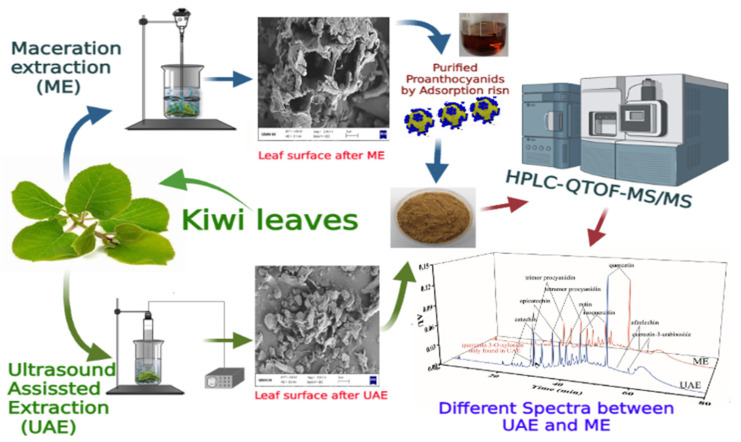
Flowchart of proanthocyanidins (PAs) extraction by ultrasound-assisted extraction (UAE) and Maceration extractrion (ME) methods.

Fresh Kiwi (*Actinidia chinensis*) leaves were collected during October from Zhuji local farm, Shaoxing, China. The cleaned leaves, as presented in Figure 1, were freeze-dried before being ground into powder and then pass through a 0.25 mm mesh screen. Kiwi leaves’ powders were stored at −20 °C until further extraction.

### 2.2. Extraction of PAs

#### 2.2.1. Ultrasound-Assisted Extraction (UAE)

The ultrasound-assisted extraction technique was performed according to Martin-Garcia et al. [27]. In brief, 1 g of kiwi leaves’ freeze-dried powders were mixed with an appropriate volume of acetone solvent (10–70 mL/g (S/S)) in 3.5 cm inner diameter cylindrical glass. UAE protocol was conducted by using different sonication power (0–70% US-amplitude), temperature (0–30 °C), and time of ultrasonic bath system (JY92-IIDN, Ningbo Scientz Biotechnology Co., Ningbo, China). In addition, acetone was used as an ideal solvent for PA extraction from plants [27]. The supernatant was filtered by syringe filters (nylon membrane, pore size 0.45 μm) and then freeze-dried to obtain the PA crude extracts. The crude extracts were stored at 4 °C for further analysis.

#### 2.2.2. Maceration Extraction (ME)

In order to compare the PAs extraction efficiency of UAE with the conventional methods, ME was carried out as described by Xu et al. [28] with slight modifications. An aliquot of 10 g of freeze-dried powder was mixed with 300 mL acetone solution (70%, *v/v*) at 30 °C and stirring at 200 rpm for 1 h. The extracted solution was filtered, freeze-dried, and stored at 4 °C in the same way as UAE. 

### 2.3. Purification of the Extracted PAs 

Crude extracts obtained in both UAE and ME were purified following Luo et al. [29]. In brief, the crude extracts (1 g) were loaded onto AB-8 Macroporous resin column and washed with distilled water (4 times column volume) to remove impurities, such as sugar, proteins, and pigments. Subsequently, 4 times column volume of ethanol solution (70%, *v/v*) was used to elute the PAs and the fraction filtered. Then, the extracts were lyophilized to remove the solvent and to obtain the PKLPs. Figure 1 presents the flowchart of proanthocyanidin extraction by ultrasound-assisted extraction (UAE) and Maceration extractrion (ME) methods. PA yield (%) was calculated by the following equation:

(1) $$\mathrm{PAs}\;\mathrm{yield}\;(\%)=\frac{dry\;weight\;of\;purified\;proanthocyanidins\;powders}{dry\;weight\;of\;kiwifruit\;leaves\;powders}$$

### 2.4. Experimental Design

#### 2.4.1. Single-Factor Experiments

Single-factor experiments were employed to determine the factors resulting in response values that were very close to the optimum region [30]. The response variable (Y) of extracted PAs was influenced by each independent parameter. In this part, the initial range of four designed factors were investigated including US amplitude (0–60%), sonication time (0–25 min), temperature (10–70 °C), and solvent to solid (S/S) ratio (10:1–70:1 mL/g).

#### 2.4.2. Response Surface Methodology (RSM) Experiments

Based on the single-factor experimental results, the RSM was used to optimize the extraction of PAs, and its purpose was to explore the optimum condition to maximize the extraction efficiency of PAs. A central composite design (CCD) with four-factor and five-level was conducted for the best model due to its accurate prediction and economic approach [30,31]. In that model, US amplitude (X_1_, %), sonication time (X_2_, min), temperature (X_3_, °C), and solvent to solid ratio (X_4_, mL/g) were the independent variables, and their coded and uncoded levels were displayed in Table 1. Additionally, 30 experimental runs and 6 replicates of the center point were used to optimum the extraction model. In addition, the relationship between the four independent variables and the extraction yield of PAs was expressed as a second-order polynomial equation:

(2) $$Y=\alpha_{0}+\sum_{i=1}^{n}\alpha_{i}X_{i}+\sum_{i=1}^{n}\alpha_{\mathrm{ii}}X_{i}^{2}+\sum_{i=1}^{n}\sum_{j=1}^{n}\alpha_{\mathrm{ij}}X_{i}X_{j}$$

where Y is the predicted value of extraction yield of PAs, α_0_ is a constant; α_i_, α_ii_, and α_ij_ are the linear, quadratic, and interactive regression coefficients of the model, respectively; X_i_ and X_j_ are the independent variables.

3D surface plots were constructed to exhibit the interactive effects between independent factors, which enabled the visualization of the relationships between the variables in the plot and the response.

### 2.5. Experimental Methods

#### 2.5.1. Determination of Total Phenolics Content (TPC) and PAs Content and In Vitro Antioxidant Activity

Total phenolics were determined spectrophotometrically by using the Folin–Ciocalteu method following Gouda et al. [32] with minor modifications. The TPC of the extracts was expressed as mg of catechin (CAT) equivalents per g of sample.

The PA content was measured using the vanillin method described by Cao et al. [13]. Briefly, 0.5 mL of sample was mixed with 2.5 mL vanillin (1% in methanol, *v/v*) followed by 2.5 mL of H_2_SO_4_ (20% in methanol, *v/v*). The absorbance was measured at 500 nm after incubation at 30 °C for 20 min. Results were expressed on a dry weight basis (DW) as mg procyanidin (PC) equivalent per g of sample.

Diphenyl-1-picrylhydrazyl (DPPH) radical-scavenging activity and antioxidant power (ABTS) assays were employed to evaluate the antioxidant activity [1]. In brief, freeze-dried PKLPs were dissolved in phosphate buffer solution (PBS), and then 20 μL of the PKLPs (0–0.05 mg/mL) was mixed with 200 μL of methanolic solution of DPPH (0.04 mg/mL) and left for 30 min in the dark. The absorbance (Abs) was measured at the 517 nm wavelength using the EPOCH2 microplate reader (BioTek, Inc., Winooski, VT, USA).

The IC_50_ values were calculated by using a regression equation between the concentration and the antioxidant percentage of each sample.

The antiradical power was measured using the 2.2′-azino-bis-3-ethylbenzothiazoline-6-sulphonic acid (ABTS) radical scavenging modified method of Gouda et al. [32]. Briefly, the ABTS stock solution (7 mM) was mixed with an equal volume of potassium persulfate (2.45 mM), then incubated at 4 °C for 16 h to produce ABTS radical cation (ABTS^•+^). The ABTS^•+^ solution was diluted with distilled water to maintain an absorbance of 0.70 ± 0.01 at 734 nm. Then, 20 μL of 0–0.05 mg/mL PKLPs in PBS was mixed with 3 mL of ABTS^•+^ solution and kept in a dark place for 6 min at 25 °C before the measurement. The Abs was measured at 734 nm wavelength using an EPOCH2 microplate reader (BioTek, Inc., Winooski, VT, USA). IC_50_ was measured using the same formula of the DPPH method. All of the measurements were performed in three replicates.

#### 2.5.2. HPLC-QTOF-MS/MS Analysis

Separation and structure identification of PKLPs was conducted using a reversed-phase HPLC system (Waters e2695, Waters Corp., Milford, MA, USA) following the method of Huang et al. [33] with minor modifications. The system included a quaternary pump coupled UV-Vis detector (Waters 2489, Waters Corp., Milford, MA, USA) and equipped with Agilent Zorbax Extend-C18 column (4.6 × 250 mm, 5 μm, (Agilent Tech Co., City, CA, USA). In brief, the injected volume was 10 μL, 35 °C with the flow rate of the mobile phase fixed at 0.7 mL/min, and the detection wavelength was set at 280 nm to monitor all phenolic compounds. Two elution solvents, A (water: formic acid; 99.7: 0.3, *v/v*) and B (acetonitrile), were used with the following gradient elution program: 3% B (0 min); 3–25% B (0–56 min); 24–67% B (56–79 min); 100% B (79–80 min).

MS spectra were recorded on a Triple-TOF 5600+ ion trap mass spectrometer (AB scientific, Framingham, MA, USA). The mass spectrometer was operated in negative ion mode using the following conditions: 50–1500 *m/z* mass range, 10 V capillary voltage, 550 °C capillary temperature, 4.5 kV ion spray voltage, 35 arb sheath gas (N_2_), 6 arb auxiliary gas (N_2_), and 80 V tube lens offset voltage.

#### 2.5.3. Cell Culture and Treatment

Caco-2 (human colorectal adenocarcinoma) cell lines were provided by Zhejiang Key Laboratory for Agro-Food Processing (Zhejiang University, Hangzhou, China). The cells were cultured at 37 °C, in Dulbecco’s modified eagle medium (DMEM) medium with 20% Fetal Bovine Serum (FBS) and 1% penicillin-streptomycin (P/S) under 5% CO_2_. Cells were subcultured 3–4 times per week by replacing fresh medium to keep the cells in a good growth state.

#### 2.5.4. Cytotoxicity Analysis

The cytotoxicity effect of extracted PAs on Caco-2 cells were tested following Gao et al. [34] with some modification. In addition, 100 μL of Caco-2 cells was seeded into 96-well plates at a density of 2 × 10^5^ cells/well and incubated for 48 h in standard cell culture condition before being replaced by fresh medium. Then, 10 μL of samples with various concentrations (0–125 μg/mL) were added to each well, and the control was treated without a sample solution; the blank wells only contain growth medium. After incubating for 24 h, 20 μL of MTT (0.5 mg/mL) was added, and the plates were incubated 4 h at 37 °C under 5% CO_2_. Afterwards, the medium was removed, and 100 μL of DMSO was added to dissolve the MTT-formazan complex. The absorbance of the cells was measured at 570 nm and compared to the control. The IC_50_ values were calculated after plotting cell viability versus reagent concentration.

#### 2.5.5. Antioxidant Activity on H_2_O_2_-Induced Cell Death in Caco-2 Cells

##### Injured Cell Model Induced by H_2_O_2_

The antioxidant activity on H_2_O_2_-induced cell death on Caco-2 cells was determined according to Cilla et al. [26]. Briefly, 5 mL medium contains 2 × 10^6^ cells/well was pipetted into 6-well plate (Costar Corning, Rochester, NY, USA), and incubated at 37 °C under 5% CO_2_ for 24 h. Then, serial dilutions (0–250 μg/mL) of H_2_O_2_ multiple times (0–5 h) were added to each well to determine the IC_50_ concentration of H_2_O_2_ in the injured Caco-2 cells, which aim to establish the H_2_O_2_-induced oxidative injury model. The results were expressed as cell viability measured by the MTT assay.

##### Intracellular Antioxidant Activity Assay

The effect of extracted PAs’ antioxidant activity on the Caco-2 cells was measured according to Liang et al. [35]. Briefly, 5 mL medium containing 2 × 10^5^ cells/well was pipetted into 96-well plates. The medium was replaced with a fresh medium after cultivation for 24 h at 37 °C. The experimental group was treated with various concentrations of samples (0–125 μg/mL, 10 μL) for 24 h before H_2_O_2_ treatment, the control group was normally cultivated, and the model group was treated with H_2_O_2_. The result was expressed as the cell viability determined by the MTT assay.

### 2.6. Statistical Analysis

Experiments were conducted in triplicate and the average values ± standard deviation (SD) are tabled. Data were further analyzed via one-way analysis of variance (ANOVA), using SPSS 19.0 (Chicago, IL, USA). Analysis of variance (ANOVA) and correlation efficient (r^2^) were applied among all the measurements, *p* ≤ 0.05 was considered statistically significant or correlated, and *p* ≤ 0.01 was considered as highly significant. Tukey’s HSD test and Least significant differences (LSD) have been calculated to measure the signification among the tested groups and properties. In addition, IC_50_ was calculated based on the regression equation.

## 3. Results

The optimization of the UAE method on the functionality of the extracted PAs from kiwi leaves is important for further studying their exact potential bioactivity. This is because US shows high potential in the field of herbal science due to its high ability to generate better yield and low scale-up finance compared to the other emerging techniques like a microwave (MW) and pulsed electric fields (PEF) [36]. On the other hand, special attention should be focused on the chemical composition and bioactivity of extracted PAs [4]. Thus, this study conducted a single-factor experimental design to show the optimum of four significant US factors represented in power, duration, temperature, and solvent to solid ratio that have significant influences on PA extraction.

### 3.1. Single-Factor Experimental Analysis

**Figure 2:**
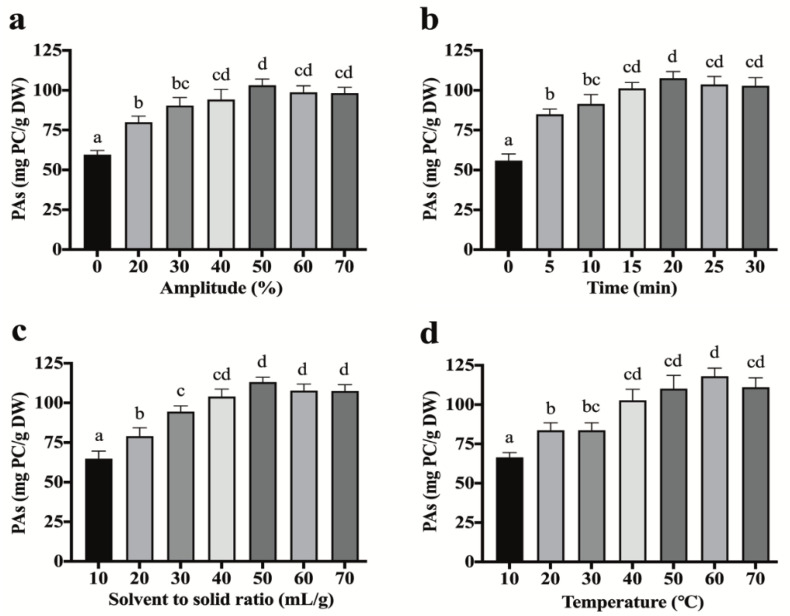
Single factor experiments of the UAE parameters’ effects on yield of PAs extracted from kiwifruit leaves: (**a**) ultrasonic amplitude; (**b**) extraction time; (**c**) solvent to solid ratio; and (**d**) extraction temperature. Mean ± SD with different alphabet superscript within the same column and analytical parameter indicate that values differ significantly (*p* < 0.05).

Single-factor experiments were designed to evaluate the influences of the four US-related factors (amplitude, sonication time, extraction temperature, and S/S ratio) on PA extraction yields (Figure 2). These experiments presented four different factors that could affect the extraction efficiency of PAs from kiwi leaves.

### 3.2. Effect of US Amplitude on PA Extraction

In order to improve the US-assisted efficiency of extracted PAs from kiwifruit leaves, ultrasonic amplitude (0, 20, 30, 40, 50, 60, 70%) was carried out. Other parameters were at fixed conditions (15 min, 30 °C, and 30 mL/g S/S ratio). A significant (*p* < 0.05) increase in PA yield (79.90 ± 3.91 mg PC/g DW) after 15 min, and the highest extracted yield was obtained at 50% (103.12 ± 3.93 mg PC/g DW) compared to the control with 59.58 ± 2.67 mg PC/g DW (Figure 2a). Then, reverse effects have happened from 60% (98.69 ± 4.09 mg PC/g DW), but the differences did not attend the significant level (*p* > 0.1). This phenomenon could be explained by the potential increase in the cavitation bubbles by an increase in ultrasonic power, which increases the solvent intracellular transfer. A US mechanophore breakage (mechanochemical breakage of the polymer reactive units like cyclic rings) can occur in extracts’ solutions via the shear stress caused by the collapse of US-induced cavitation bubbles [37,38]. These modifications in the structure of extracted compounds caused by US can facilitate the bioavailability of the bioactive molecules extracted from plants [16]. However, the US higher than 50% had a negative effect on the PAs due to the formed radicals and H_2_O_2_ by the cavitation’s bubbles [4]. Therefore, 50% amplitude was selected as the optimum used power for the extraction of PAs. Zhao et al. [39] reported that high US-amplitude (600 W) significantly degraded the extracted phytochemicals.

### 3.3. Effect of Sonication Time on PAs

To optimize the extraction capacity of UAE for the recovery of PAs, various ranges of sonication time (0, 5, 10, 15, 20, 25, 30 min) were evaluated. Other parameters were fixed at 30% US amplitude, 30 °C temp., and 30 mL/g S/S ratio. It has been observed that the yield of PAs was increased significantly (*p* < 0.05) with sonication time of up to 20 min (107.51 ± 4.28 mg PC/g DW) compared to the control with 55.82 ± 4.32 mg PC/g DW. Then, a slight decrease happened in a longer duration (25, 30 min). Patil and Akamanchi [40] used US (20 kHz, 150 W, 30 °C) for extraction of camptothecin from *Nothapodyhtes nimmoniana* leaves. They mentioned that the application of US increased the camptothecin yield (1.7-fold) and decreased the extraction time from 6 h to 18 min. This could be due to the potential impact of US to transform the disulfide bonds to thiol bonds of the molecules, which results in changing the covalently attached linear polymer chains in the *β*-position to a disulfide moiety [41]. However, long-time ultrasonic cavitation could result in the degradation of polyphenolics that declined the extraction yield [4]. Therefore, 20 min was selected as a suitable US time range for PAs.

### 3.4. Effect of Sonication Temperature on PAs

The effect of extraction temperature on PAs was performed from 10–70 °C with other fixed parameters (30% ultrasonic amplitude, 15 min sonication time, and 30 mL/g S/S ratio). The experimental results showed that, by raising the temperature of extraction from 10 to 60 °C, the yield of PAs was significantly increased from 66.48 ± 3.06 to 118.03 ± 5.20 mg PC/g DW. Then, the PA yield decreased upon further increasing of the temperature (Figure 2d). Additionally, the effect was similar to the increase in US-amplitude and time. These results might be related to the decrease of surface tension and viscosity of the solvent, which induced an increase in vapor pressure. With the increment of temperature, the sonochemical influents caused by the collapse of cavitation bubbles decreased, and polyphenols might be degraded at a higher temperature situation [4]. Therefore, controlling the temperature at an appropriate range is very necessary during the extraction process of bioactive compounds from plant materials, for which the extracted PAs by US can show a higher antioxidant capacity against DPPH, and hydroxyl radicals compared to heat reflux extraction based on the significant differences in the extracted chemical structure [42]. In addition, Huo et al. [41] reported that US mechanochemistry could decrease the exposure temperature at the molecular level by rearranging or cleaving bonds at predetermined breaking sites that could assist in facilitating the release of PAs in the lower temperature compared to the maceration method [43].

### 3.5. Effect of Solvent to Solid Ratio during the Sonication Process on PAs

To maximize the extraction efficiencies, various S/S ratios (10, 20, 30, 40, 50, 60, 70 mL/g) were selected with other fixed parameters (30% US amplitude, 15 min, and 30 °C). As presented in Figure 2c, the yield of PAs increased significantly (*p* < 0.05) at 50 mL/g (113.03 ± 3.10 mg PC/g DW) compared to 10 mL/g with 64.88 ± 4.83 mg PC/g DW. After reaching a peak, the yield of PAs slightly decreased when the S/S ratio further increased above 50 mL/g. A possible explanation is that the US cavitation effect could decrease the concentration of the high S/S ratio level by improving the rates of heat and mass transfer, cell disruption, and the penetration of solvents to the herbal tissues [13,44]. Xu et al. [21] reported that 40 mL/g of S/S was the most efficient solvent concentration, which improved the solubility of PAs in the plant cells. Boulatov [16] reported that the US mechanochemistry affected overstretching carbohydrates’ polymers that lead to their fragmentation, which helps in releasing small molecules that are bound in their polymer chain.

### 3.6. Analysis of Response Surface Methodology

#### 3.6.1. Model Fitting

The experimental data of extracted PAs from kiwi leaves ranged from 70.59 to 122.19 mg PC/g DW, and TPC ranged from 105 to 177.15 mg CAT/g DW (Table 1). They were optimized based on the CCD and evaluated by the linear regression and ANOVA (Table 2). In addition, the applicability of the model is typically dependent on the realization of the significant regression coefficient (R^2^) and insignificant lack of fit [45,46]. The importance of measuring TPC with PAs was due to their strong correlations, as PAs belong to TPC [14], with the possibility of selecting the conditions with the highest concentration of TPC (177.15 mg CAT/g DW), and, as a consequence, stronger validation of PA extracts with 122.19 mg PC/g DW at the same condition of 40% (US-amplitude), 15 min, 30 mL/g (S/S ratio), and 70 °C (Table 1). Leontowicz et al. [47] mentioned the strong correlation between TPC and flavanols in kiwi fruit. This can indicate the suitability of the used extraction conditions of PAs, as flavan-3-ols, through its verification by total phenolic contents.

**Figure 3:**
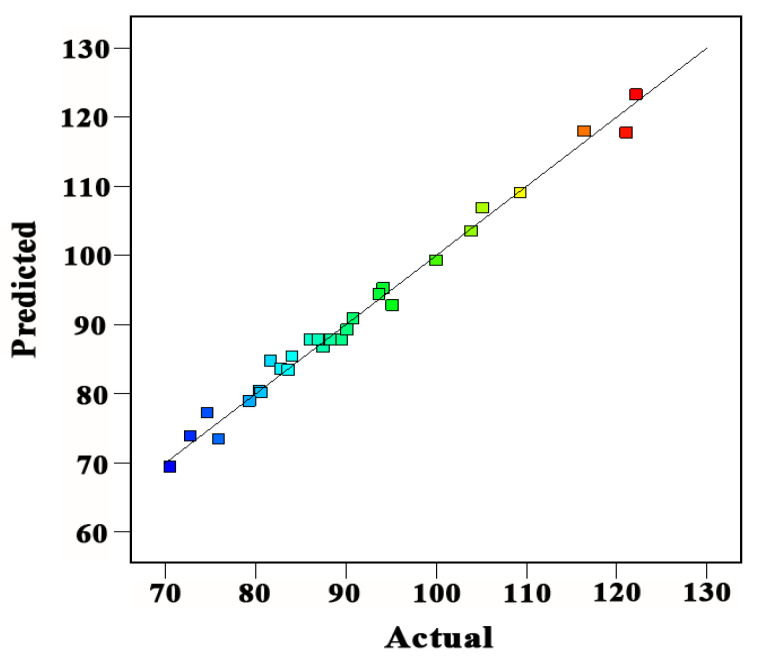
Correlation between the actual and predicted values of extracted yield of proanthocyanidins (PAs) from kiwifruit leaves.

In this study, R^2^ was highly significant (R^2^ = 0.9882) and the lack of fit was insignificant (*p*-value = 0.0771), which indicates that the prediction model was valid for PA extraction. Meanwhile, there are adjusted and predicted coefficients ($R_{\mathrm{adj}}^{2}=0.9772$ and $R_{\mathrm{pre}}^{2}=0.9381$) and the high-value Adeq. precision (37.40) with a low value of the coefficient of variation (C.V. = 2.24%), which indicated a high reliability of experimental data with a very high degree of precision (Figure 3). Esua et al. [48] mentioned that C.V. values < 10% for responses suggesting the reliability, precision, and reproducibility of the established experiments. Therefore, the current analysis showed that both mathematical models for calribration and prediction were acceptable in describing the results of US-assisted extraction for kiwi leaves. In addition, this study experimental model agreed with Ismail et al. [49], who reported that the coefficient of variation (C.V. < 0.2445) indicates a low variation in the mean values and a higher degree of precision and reliability.

#### 3.6.2. Interaction of Independent Variables on Extraction PAs in the RSM Model

As presented in Table 2, the linear regression coefficients of US-amplitude (X_1_); extraction time (X_2_); S/S ratio (X_3_); and extraction temperature (X_4_) were significant (*p* < 0.05). However, X_1_X_2_, X_2_X_3_, X_2_X_4_, $X_{1}^{2}$, $X_{2}^{2}$, and $X_{3}^{2}$ were insignificant (*p* > 0.05), which indicates a highly significant effect on the surface response [50]. According to experimental results, the final predictive second-order polynomial equation describes the effectiveness of extracted PAs by UAE, taking into consideration only the significant parameters, which was presented in the following equation:

(3) $$Y\;=\;105.39\;+\;0.83X_{1}-0.78X_{2}-0.42X_{3}-2.82X_{4}-0.01X_{1}X_{3}-0.01X_{1}X_{4}+0.03X_{3}X_{4}+0.03X_{4}^{2}$$

where Y represents the yield of PAs; X_1_, X_2_, X_3_, and X_4_ are the coded variables for the ultrasonic amplitude, ultrasonic time, S/S ratio, and extraction temperature, respectively.

#### 3.6.3. Response Surface Methodology of the Four US Affected Factors’ Variables

**Figure 4:**
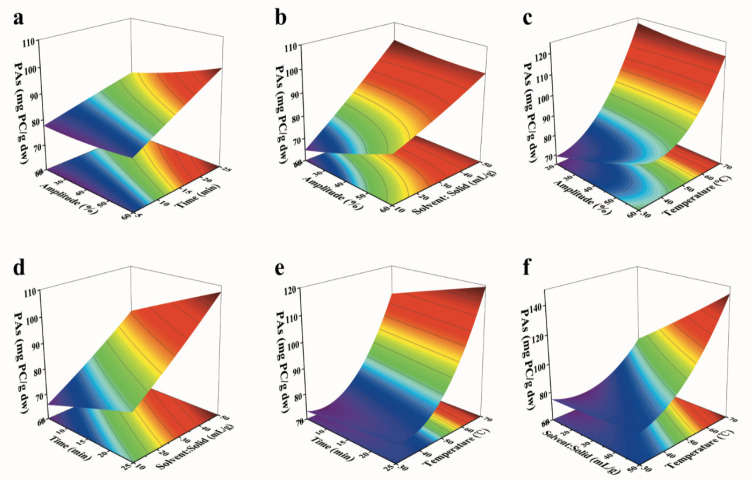
Response surface methodology (RSM) for the ultrasound-assisted extraction (UAE) of extracted yield of proanthocyanidins (PAs) from kiwi leaves with respect to ultrasonic amplitude (**X1**); extraction time (**X2**); solvent to solid ratio (**X3**); and extraction temperature (**X4**). (**a**) RSM between amplitude and time; (**b**) RSM between amplitude and solvent to solid ratio; (**c**) RSM between amplitude and temperature; (**d**) RSM between time and solvent to solid ratio; (**e**) RSM between time and temperature; (**f**) RSM between solvent to solid ratio and temperature.

Three-dimensional response surface and contour plots (Figure 4) were used to illustrate the interactive effects between four independent variables and the extraction yield of PAs [51]. The PAs (*z*-axis) were plotted against two independent variables while keeping the rest of the variables at a fixed level. Figure 4a displayed the interactive effect between US-amplitude and time on the yield of PAs with the S/S ratio and extraction temperature at a fixed value. The yield of PAs was significantly increased with the exposure time, and it was slightly increased with increasing US-amplitude. The best explanation for this phenomenon is that temperature from the higher US-amplitude is increasing the mass transfer, facilitating the release of molecules from the leaves’ tissues [4]. An increase of S/S from 10 to 50 mL/g increased the extraction yield of PAs significantly, while the extraction yield of PAs increased slightly with the enhancement of ultrasonic amplitude from 20% to 60% (Figure 4b). On the other hand, the values of PAs increased dramatically with increasing the extraction temperature, reaching the optimum level at 50–70 °C (Figure 4c). The temperature had a significant effect on extraction while the influence of US-amplitude on the response did not attend a significant level (*p* > 0.1) since temperature had a dual effect on both solvent and solute. Guo et al. [52] mentioned that the adsorption ratio of the flavonoids was negatively correlated with the temperature. PAs as flavons are facilitating their release from leaves by increasing temperature. The PAs were shown in Figure 4d as the interactive influence on ultrasonic time and S/S ratio. PAs increased rapidly as the function of S/S ratio, while the ultrasonic time had a slight effect on the extraction of PAs. The highest values of PAs were observed at a longer extractive time and higher S/S ratio. Meanwhile, the maximum extraction of PAs could be obtained at a longer extractive time and higher temperature with other extraction parameters being fixed (Figure 4e). Ismail et al. [49] reported that US for 20 min and 30% US-amplitude was the optimum condition for PA yield. In that study, the higher amplitude during ultrasonic irradiation resulted in the oxidization of PAs, which caused the decline of extraction yield. Moreover, the interactive effect of S/S ratio and temperature demonstrated a significant interrelation between both influencing factors (Figure 4f). According to the response model, the PA yield increased with an increase in S/S ratio and temperature until 50 mL/g and 70 °C as the highest condition.

The final optimum UAE conditions were concluded as follows: 40% (US-amplitude), 15 min, 30 mL/g (S/S ratio), and 70 °C. Meanwhile, the experimental yield of PAs was close to the predicted yield (confidence level > 98%)—in which, under the optimal UAE conditions, PA experimental values were 122.19 mg PC/g DW, which was matched well with the predicted value (119.55 mg PC/g DW).

### 3.7. Comparison between Ultrasound-Assisted Extraction (UAE) and Traditional Maceration Extraction (ME) Efficiency and Identification of PA Fractions

In order to validate the effectiveness of ultrasound on the extraction of PAs from kiwi leaves, a comparison was carried out between UAE and ME. ME is considered a common technique that has been employed numerous times by different researchers for the extraction of polyphenol and PAs [27,53,54]. It was observed that UAE significantly increased the extracted PA yield (11.3 ± 1.41%) compared to the maceration method (8.65 ± 1.2%) for which ABTS and DPPH showed that the antioxidant activity of ultrasound-assisted extracts was significantly (*p* < 0.05) higher than ME (Table 3).

**Figure 5:**
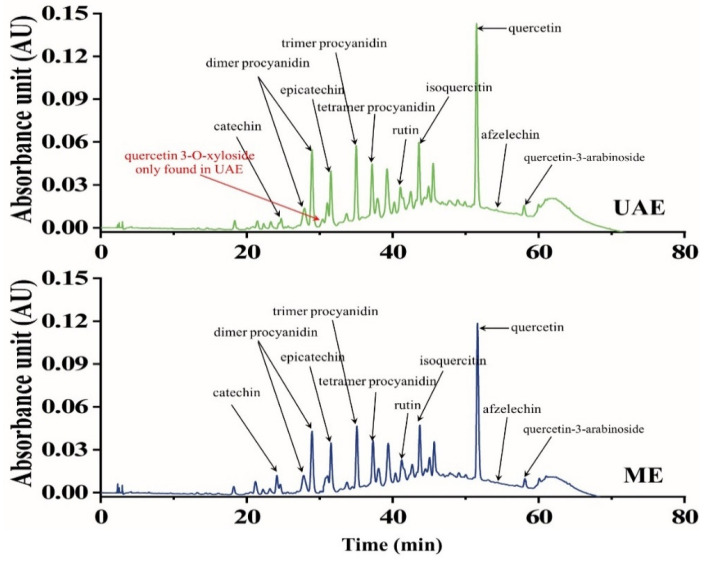
HPLC-QTOF-MS/MS spectra of the chemical composition differences between ultrasound-assisted extraction (UAE) and maceration extraction (ME) methods.

Additionally, the fraction of phenolics by HPLC-QTOF-MS/MS showed a higher relative percentage of several sensitive compounds like benzoyl glucuronide, catechin, and isoquercitin in UAE compared to ME (Table 4; Table S1; Figure 5). Meanwhile, quercetin 3-O-xyloside was found in UAE, which means that the validation of the optimum condition in extracting PA compounds can enhance the efficiency of the separated phytochemicals.

**Figure 6:**
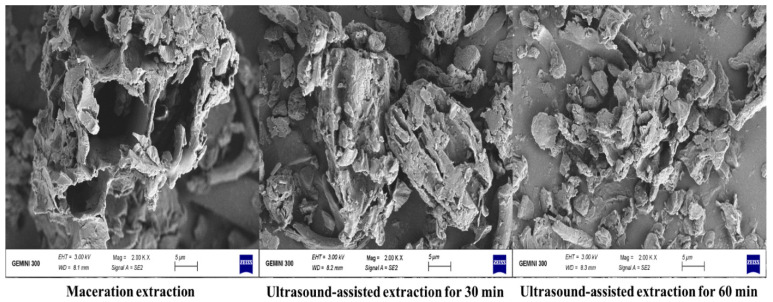
Scanning electron micrographs of ultrasound-assisted extraction (UAE) and Maceration extraction (ME) methods.

In addition, a scanning electron microscope showed high differences between using UAE and ME through an obvious degradation of leaves’ fibers microstructure by US after 30 min and 60 min compared to maceration (Figure 6).

These differences in intramacromolecules’ connective tissues facilitated the release of PAs in US-treated samples. Similar observations have been reported for US treatment by Esua, Cheng, and Sun [48]. In addition, Pudziuvelyte et al. [55] reported that US significantly increased the extracted phenolic yield (855.54 μg/g) of *lsholtzia ciliata* leaves compared to the maceration method (141.06 μg/g) through microstructure degradation. They mentioned that US for 11 min increased the mass fraction of total phenols by 20% compared to water bath shakers for 30 min with the same solvent.

### 3.8. Evaluation of Bioactive Functionality of the Extracted PAs by Cytotoxicity and Cellular Antioxidant Assays

**Figure 7:**
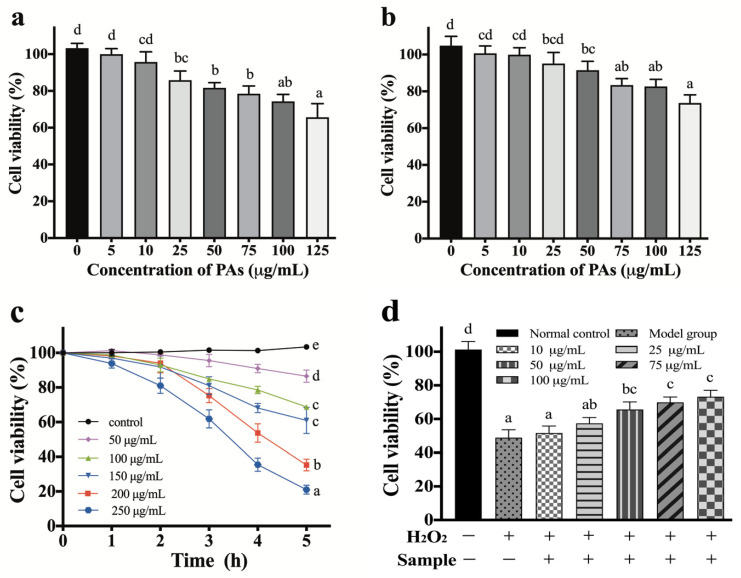
Cytotoxicity analysis of PKLPs by using various cells. (**a**) Caco-2 cells; (**b**) HepG-2 cells; (**c**) cell viability effect by various concentration of H_2_O_2_ (0–250 μg/mL); and (**d**) antioxidant potential of PKLPs on the H_2_O_2_-induced Caco-2 cells model after 24 h of incubation. Mean ± SD with different alphabet superscripts within the same column and analytical parameter indicate that values differ significantly (*p* < 0.05).

Due to the global concern of cancer treatment by natural safe bioactive molecules [22], in this study, the extracted Pas’ functionality has been evaluated. The cytotoxicity anticancer activity through seven concentrations (5, 10, 25, 50, 75, 100, 125 µg/mL) of PAs against HepG-2 and Caco-2 cell line revealed the high efficiency of the extracted PAs by optimized conditions of UAE. A marked reduction in the probable cancer risk was observed for the exposure against the two species (Figure 7), and there was a significant reduction (*p* < 0.01) in Caco-2 cell viability after 25 µg/mL (Figure 7a). Meanwhile, the significant reduction (*p* < 0.05) in HepG-2 cell viability was after 50 µg/mL. Moreover, Caco-2 was more sensitive to PAs compared to HepG-2. Kumari and Gupta [51] reported a marked reduction in probable cancer under RSM optimized conditions. In addition, the mechanistic and preclinical studies demonstrated that the capacity of PAs to modulate the several factors related to colorectal cancer is based on its polymerization degree [56]. Leontowicz et al. [47] mentioned that high polyphenolic and flavonoids, flavanols, and tannins’ contents of kiwi have a very significant inhibition impact on the growth of cancer cells.

In addition, H_2_O_2_ is a typical member of the ROS family, which were wildly used to construct the oxidative injury model to evaluate the natural extracted PA effect on anticancer cells [57]. As showed in Figure 7c, the cell viability decreased dramatically (*p* < 0.05) when exposed to the highest concentration of H_2_O_2_ (250 μg/mL) compared to the low concentration of H_2_O_2_ (0–100 μg/mL). In particular, 53.69 ± 7.29% treated cells were viable after being treated with 100 μM of H_2_O_2_ for 4 h at 37 °C. As this value was close to the IC_50_, this indicated that the H_2_O_2_-injured-Caco-2 model was established. Additionally, for the antioxidant assay, a moderate increase was found when Caco-2 cells were treated with appropriate concentrations of PAs (10–25 μg/mL) for 24 h before H_2_O_2_ treatment. Furthermore, a significant increase (*p* < 0.05) was happened when PKLP concentration increased to 100 µg/mL compared to 10 µg/mL (Figure 7d). The potential cellular redox activity might come from the increase in the reactivity of PAs with the oxidizing radicals that prevent the dissociation of both intramolecular and intermolecular disulfide bonds. This process is necessary for protein activity regulation that affects several cellular signal pathways and enzymatic reactions [58]. Additionally, the differences in PKLP cytotoxicity and cytoprotection were due to their dose dependent impact on the cellular pathways of the same cells [59], in which only at the lowest concentrations was a slight cytoprotection assayed. Meng et al. [60] reported that the cellular antioxidant activity is one of the best methods to explain the real redox homeostasis impact of natural phytochemicals that benefit human health.

## 4. Conclusions

In this study, the optimum US conditions on the extraction of bioactive and functional PAs were demonstrated. The four most important factors which affect the extraction of PAs and their potential interactions were evaluated to optimize their conditions. In conclusion, the characterization of PKLPs could be used as a functional food ingredient considering its potent antioxidant potential activity, which would have broad prospects and substantial economic benefits. The relationship between structure and bioactivity of PKLPs warrants further studies. This investigation is providing important information on the correlation between the potential impact of the extraction method on the formation and modifications of extracted PAs.

## Figures and Tables

**Table 1:** Response surface design with experimental and predicted results.

Run	Factor X1 Amplitude % (Code)	Factor X2 Time Min (Code)	Factor X3 Solvent/Solid mL/g (Code)	Factor X4 Temp. °C (Code)	PAs (mg PC/g DW)		TPC (mg CAT/g DW)	
					Experimental	Predicted	Experimental	Predicted
**1**	30 (−1)	10 (−1)	40 (+1)	60 (+1)	109.36 ± 7.23	105.81	162.11 ± 6.01	161.50
**2**	40 (0)	15 (0)	30 (0)	50 (0)	87.32 ± 5.15	85.01	135.69 ± 5.67	132.36
**3**	30 (−1)	10 (−1)	20 (−1)	60 (+1)	87.54 ± 4.87	84.45	131.92 ± 4.17	132.14
**4**	40 (0)	5 (−2)	30 (0)	50 (0)	80.65 ± 5.1	77.69	125.18 ± 3.69	123.71
**5**	40 (0)	15 (0)	30 (0)	70 (+2)	122.19 ± 5.71	119.55	177.15 ± 7.79	176.86
**6**	40 (0)	15 (0)	30 (0)	50 (0)	89.54 ± 3.57	85.01	127.91 ± 3.89	132.36
**7**	40 (0)	25 (+2)	30 (0)	50 (0)	94.18 ± 3.48	92.13	141.44 ± 7.70	144.40
**8**	40 (0)	15 (0)	50 (+2)	50 (0)	103.89 ± 4.18	99.67	141.37 ± 4.07	140.83
**9**	50 (+1)	10 (−1)	20 (−1)	60 (+1)	90.19 ± 5.08	86.29	133.61 ± 3.79	132.22
**10**	30 (−1)	20 (+1)	20 (−1)	40 (−1)	75.96 ± 3.52	71.63	120.24 ± 3.34	117.37
**11**	50 (+1)	20 (+1)	20 (−1)	40 (−1)	82.87 ± 4.11	81.07	138.44 ± 4.74	137.29
**12**	20 (−2)	15 (0)	30 (0)	50 (0)	81.73 ± 4.57	82.57	129.46 ± 4.77	132.58
**13**	50 (+1)	10 (−1)	40 (+1)	60 (+1)	105.18 ± 5.67	102.85	159.67 ± 3.93	158.42
**14**	40 (0)	15 (0)	30 (0)	50 (0)	86.99 ± 4.25	85.01	137.67 ± 5.15	132.36
**15**	50 (+1)	10 (−1)	20 (−1)	40 (−1)	74.68 ± 3.81	74.95	117.81 ± 2.95	115.55
**16**	50 (+1)	20 (+1)	40 (+1)	60 (+1)	116.42 ± 6.23	113.57	169.71 ± 5.41	168.57
**17**	50 (+1)	10 (−1)	40 (+1)	40 (−1)	83.67 ± 4.52	80.31	124.36 ± 3.72	128.91
**18**	30 (−1)	20 (+1)	40 (+1)	40 (−1)	84.08 ± 4.61	82.79	125.28 ± 3.35	124.91
**19**	30 (−1)	20 (+1)	40 (+1)	60 (+1)	121.11 ± 3.08	114.13	162.37 ± 5.73	160.45
**20**	40 (0)	15 (0)	10 (−2)	50 (0)	72.82 ± 4.06	71.95	105.06 ± 4.13	107.09
**21**	30 (−1)	20 (+1)	20 (−1)	60 (+1)	93.75 ± 4.35	91.77	145.66 ± 3.94	139.71
**22**	40 (0)	15 (0)	30 (0)	50 (0)	86.12 ± 3.56	85.01	137.27 ± 2.52	132.36
**23**	30 (−1)	10 (−1)	20 (−1)	40 (−1)	70.59 ± 4.11	67.91	107.45 ± 4.58	106.83
**24**	30 (−1)	10 (−1)	40 (+1)	40 (−1)	80.45 ± 4.19	78.07	127.30 ± 4.18	122.99
**25**	40 (0)	15 (0)	30 (0)	50 (0)	88.23 ± 5.07	85.01	127.13 ± 4.32	132.36
**26**	60 (+2)	15 (0)	30 (0)	50 (0)	95.19 ± 4.23	89.05	150.96 ± 4.48	149.42
**27**	40 (0)	15 (0)	30 (0)	50 (0)	88.37 ± 5.15	85.01	132.66 ± 3.95	132.36
**28**	40 (0)	15 (0)	30 (0)	30 (−2)	79.37 ± 4.51	76.87	122.94 ± 4.17	124.65
**29**	50 (+1)	20 (+1)	40 (+1)	40 (−1)	90.87 ± 4.81	87.43	146.06 ± 2.99	141.67
**30**	50 (+1)	20 (+1)	20 (−1)	60 (+1)	100.07 ± 6.28	96.01	150.82 ± 3.16	150.99

**Table 2:** Analysis of Variance (ANOVA), factors, and their interaction effects.

Source	Sum of Squares	df	Mean Square	F-Value	*p*-Value
**Model**	5213.5733	14	372.3981	89.90	<0.0001
**X_1_-Amplitude**	96.1200	1	96.1200	23.20	0.0002
**X_2_-Extraction Time**	341.4867	1	341.4867	82.44	<0.0001
**X_3_-Solvent: solid**	1314.6840	1	1314.6840	317.38	<0.0001
**X_4_-Temperature**	2950.1620	1	2950.1620	712.20	<0.0001
**X_1_X_2_**	5.7002	1	5.7002	1.38	0.2591
**X_1_X_3_**	22.1606	1	22.1606	5.35	0.0353
**X_1_X_4_**	27.3268	1	27.3268	6.60	0.0214
**X_2_X_3_**	1.0868	1	1.0868	0.26	0.6160
**X_2_X_4_**	13.4873	1	13.4873	3.26	0.0913
**X_3_X_4_**	129.6752	1	129.6752	31.30	<0.0001
$X_{1}^{2}$	1.6450	1	1.6450	0.40	0.5381
$X_{2}^{2}$	0.0073	1	0.0073	0.00	0.9670
$X_{3}^{2}$	1.3113	1	1.3113	0.32	0.5820
$X_{4}^{2}$	303.2210	1	303.2210	73.20	<0.0001
**Residual**	62.1349	15	4.1423		
**Lack of Fit**	54.8974	10	5.4897	3.79	0.0771
**Pure Error**	7.2375	5	1.4475		
**Cor Total**	5275.7082	29			
**R^2^**	0.9882				
**Adjusted R^2^**	0.9772				
**Predicted R^2^**	0.9381				
**Adeq. Precision**	37.40				
**Mean**	90.7793				
**C.V. %**	2.2420				

**Table 3:** Comparative yield, PAs, and chemical-based antioxidant activity of kiwifruit leaves by different extractions.

Response	Yield (%)	PAs (mg PC/g DW)	ABTS IC_50_ (μg/mL)	DPPH IC_50_ (μg/mL)
**UAE**	11.3 ± 1.41 ^b^	122.19 ± 5.71 ^b^	10.88 ± 0.31 ^a^	13.67 ± 0.39 ^a^
**ME**	8.65 ± 1.20 ^a^	93.7 ± 5.69 ^a^	13.67 ± 0.39 ^b^	17.59 ± 0.44 ^b^

Mean ± SD with different alphabet superscript within the same column and analytical parameter indicate that values differ significantly (*p* < 0.05).

**Table 4:** Extracted compositions identified by HPLC-QTOF-MS/MS.

Time (min)	Formula	Mass (*m/z*)	Compound Identification	MS/MS Fragment (*m/z*)	UAE	ME
					Relative Percent (%)	
**18.36**	C_20_H_22_O_5_	341	caffeyl glucopyranose	326; 319; 253; 225	0.862	0.844
**21.47**	C_13_H_14_O_8_	297	benzoyl glucuronide	179; 135; 297	1.749	0.903
**22.33**	C_30_H_26_O_13_	593	dimer propelargonidins	441; 467; 425	0.822	0.597
**23.31**	C_21_H_22_O_12_	465	taxifolin hexoside	285; 179; 301	0.461	0.343
**24.72**	C_15_H_14_O_6_	289	catechin	289; 181; 137; 125; 151	2.137	1.048
**27.91**	C_30_H_26_O_12_	577	dimer procyanidin	577; 449; 425; 289; 287	3.399	3.135
**28.95**	C_30_H_26_O_12_	577	dimer procyanidin isomer	577; 449; 425; 289; 287	9.022	8.308
**30.39**	C_20_H_18_O_11_	433	quercetin 3-O-xyloside	325; 300; 285; 151	0.485	-
**31.54**	C_15_H_14_O_6_	289	epicatechin	289; 181; 137; 125; 151	6.77	5.68
**35.01**	C_45_H_38_O_18_	865	trimer procyanidin	577; 451; 407; 289	7.483	7.62
**37.24**	C_60_H_50_O_24_	1153	tetramer procyanidin	865; 577; 451; 289	5.949	6.044
**39.24**	C_60_H_50_O_24_	1153	tetramer procyanidin isomer	865; 577; 451; 289	8.423	6.475
**41.33**	C_27_H_30_O_16_	610	rutin	301	2.953	2.857
**45.56**	C_21_H_20_O_12_	463	isoquercitin	301; 287; 151	6.112	4.001
**51.51**	C_21_H_20_O_11_	447	quercetin	301; 271; 243; 179	18.617	17.782
**54.74**	C_15_H_14_O_5_	273	afzelechin	273; 147; 138; 126	0.305	0.212
**58.05**	C_20_H_18_O_11_	433	quercetin-3-arabinoside	300; 179	1.262	1.064

## Supplementary Materials

The following are available online at https://www.mdpi.com/article/10.3390/antiox10081317/s1, Table S1. Extracted compositions identified peaks area by HPLC-QTOF-MS/MS.

## Abbreviations

US	Using ultrasound
PAs	Proanthocyanidins
PKLPs	Purified kiwi leaves Pas
S/S	Solvent to solid ration
UAE	Ultrasound-assisted extraction
ME	Maceration extraction
RSM	Response surface methodology
CCD	Central composite design
TPC	Total phenolics content
DW	Dry weight
PC	Procyanidin
DMEM	Dulbecc’s modified Eagle medium
FBS	Foetal bovine serum
PBS	Phosphate-buffered solution
P/S	Penicillin/streptomycin
MTT	Methyl thiazolyl tetrazolium
WD	Weight basis
IC_50._	The half-inhibitory concentration
