# Supplementary material for: Ultrasound-Assisted Extraction Optimization of Proanthocyanidins from Kiwi (Actinidia chinensis) Leaves and Evaluation of Its Antioxidant Activity
Source: Antioxidants (Basel). 2021 Aug 21;10(8):1317. doi: 10.3390/antiox10081317 (PMC8389255; doi:10.3390/antiox10081317)
Supplement: Supplementary file 1 [file antioxidants-10-01317-s001.zip › antioxidants-1343287-supplementary.pdf]

antioxidants

Supplementary material

Table S1. Extracted compositions identified peaks area by HPLC-QTOF-MS/MS.

Time (min)	Formula	Mass (*m*/*z*)	Compound Identification	MS/MS Fragment (*m*/*z*)	UAE	ME
					Peak Area	
18.36	$C_{20}H_{22}O_5$	341	caffeyl glucopyranose	326; 319; 253; 225	0.110	0.097
21.47	$C_{13}H_{14}O_8$	297	benzoyl glucuronide	179; 135; 297	0.223	0.103
22.33	$C_{30}H_{26}O_{13}$	593	dimer propelargonidins	441; 467; 425	0.105	0.068
23.31	$C_{21}H_{22}O_{12}$	465	taxifolin hexoside	285; 179; 301	0.059	0.039
24.72	$C_{15}H_{14}O_6$	289	catechin	289; 181; 137; 125; 151	0.273	0.120
27.91	$C_{30}H_{26}O_{12}$	577	dimer procyanidin	577; 449; 425; 289; 287	0.434	0.359
28.95	$C_{30}H_{26}O_{12}$	577	dimer procyanidin isomer	577; 449; 425; 289; 287	1.154	0.953
30.39	$C_{20}H_{18}O_{11}$	433	quercetin 3-O-xyloside	325; 300; 285; 151	0.062	-
31.54	$C_{15}H_{14}O_6$	289	epicatechin	289; 181; 137; 125; 151	0.865	0.651
35.01	$C_{45}H_{38}O_{18}$	865	trimer procyanidin	577; 451; 407; 289	0.956	0.874
37.24	$C_{60}H_{50}O_{24}$	1153	tetramer procyanidin	865; 577; 451; 289	0.769	0.693
39.24	$C_{60}H_{50}O_{24}$	1153	tetramer procyanidin isomer	865; 577; 451; 289	1.077	0.742
41.33	$C_{27}H_{30}O_{16}$	610	rutin	301	0.377	0.327
45.56	$C_{21}H_{20}O_{12}$	463	isoquercitin	301; 287; 151	0.781	0.459
51.51	$C_{21}H_{20}O_{11}$	447	quercetin	301; 271; 243; 179	2.381	2.040
54.74	$C_{15}H_{14}O_5$	273	afzelechin	273; 147; 138; 126	0.039	0.024
58.05	$C_{20}H_{18}O_{11}$	433	quercetin-3-arabinoside	300; 179	0.161	0.122
